# Allergic patients during the COVID‐19 pandemic—Clinical practical considerations: An European Academy of Allergy and Clinical Immunology survey

**DOI:** 10.1002/clt2.12097

**Published:** 2022-01-17

**Authors:** Montserrat Alvaro‐Lozano, Mónica Sandoval‐Ruballos, Mattia Giovannini, Erika Jensen‐Jarolim, Umit Sahiner, Vesna Tomic Spiric, Cristina Quecchia, Adam Chaker, Enrico Heffler, Ludger Klimek, Helen Brough, Gunter Sturm, Eva Untersmayr, Mateo Bonini, Oliver Pfaar

**Affiliations:** ^1^ Pediatric Allergology and Clinical Immunology Hospital Sant Joan de Déu Barcelona Spain; ^2^ Childhood and Adolescence Allergic Illness Group Institut de Recerca Sant Joan de Déu Barcelona Spain; ^3^ Facultat de Medicina i Ciències de la Salut Universitat de Barcelona Barcelona Spain; ^4^ Allergy Unit Department of Pediatrics Meyer Children's University Hospital Florence Italy; ^5^ Institute of Pathophysiology and Allergy Research Center for Pathophysiology, Infectiology and Immunology Medical University Vienna Vienna Austria; ^6^ The Interuniversity Messerli Research Institute, Medical University Vienna, Veterinary University Vienna, and University Vienna Vienna Austria; ^7^ Department of Pediatric Allergy and Asthma Hacettepe University School of Medicine Ankara Turkey; ^8^ Faculty of Medicine University of Belgrade Belgrade Serbia; ^9^ Clinic of Allergology and Immunology Clinical Centre of Serbia Belgrade Serbia; ^10^ “Io e l’Asma” Center Children's Hospital ASST Spedali Civili Brescia Italy; ^11^ Department of Otolaryngology and Center of Allergy and Environment TUM School of Medicine Technical University of Munich Munich Germany; ^12^ Personalized Medicine, Asthma and Allergy Humanitas Clinical and Research Hospital IRCCS Rozzano Italy; ^13^ Department of Biomedical Sciences Humanitas University Milan Italy; ^14^ Center for Rhinology and Allergology Wiesbaden Germany; ^15^ Children's Allergy Service Evelina Children's Hospital Guy's and St. Thomas' Hospital London UK; ^16^ Paediatric Allergy Group Department of Women and Children's Health School of Life Course Sciences King's College London London UK; ^17^ Department of Dermatology and Venerology Medical University of Graz Graz Austria; ^18^ Allergy Outpatient Clinic Reummanplatz Vienna Austria; ^19^ Department of Cardiovascular and Thoracic Sciences Fondazione Policlinico Universitario A. Gemelli – IRCCS Università Cattolica del Sacro Cuore Rome Italy; ^20^ National Heart and Lung Institute (NHLI) Imperial College London London UK; ^21^ Department of Otorhinolaryngology, Head and Neck Surgery University Hospital Marburg Philipps‐Universität Marburg Marburg Germany

**Keywords:** allergen immunotherapy, allergy, biologics, COVID‐19, survey

## Abstract

**Background:**

The COVID‐19 pandemic has affected health care systems unexpectedly. However, data focusing on practical considerations experienced by health care professionals (HCPs) providing care to allergic patients is scarce.

**Methods:**

Under the framework of the European Academy of Allergy and Clinical Immunology (EAACI), a panel of experts in the field of immunotherapy developed a 42‐question online survey, to evaluate real‐life consequences of the COVID‐19 pandemic in allergy practice.

**Results:**

The respondents in the survey were 618. About 80% of HCPs indicated being significantly affected in their allergy practice. A face‐to‐face visit reduction was reported by 93% of HCPs and about a quarter completely interrupted diagnostic challenges. Patients with severe uncontrolled asthma (59%) and anaphylaxis (47%) were prioritized for in‐person care. About 81% maintained an unaltered prescription of inhaled corticosteroids (ICS) in asthmatics. About 90% did not modify intranasal corticosteroids (INCS) in patients with allergic rhinitis. Nearly half of respondents kept biological prescriptions unmodified for asthma. About 50% of respondents kept their allergen immunotherapy (AIT) prescription patterns unchanged for respiratory allergies; 60% for insect venom allergies. Oral immunotherapy (OIT) for food allergies was initiated by 27%. About 20% kept carrying out up‐dosing without modifications and 14% changed to more prolonged intervals. Telemedicine practice was increased.

**Conclusions:**

HCPs providing care to allergic patients were affected during the pandemic in diagnostic, management, and therapeutic approaches, including AIT for respiratory, insect‐venom, and food allergies. Most HCPs maintained controller treatments for both asthma, and allergic rhinitis consistent with international recommendations, as well as biological agents in asthma. Remote tools are valuable in delivering allergy care.

## 1 INTRODUCTION

The novel coronavirus outbreak referred to as “coronavirus disease 2019” (COVID‐19) caused by severe acute respiratory syndrome coronavirus 2 (SARS‐CoV‐2) infection was declared a pandemic by the World Health Organization (WHO) on March 11, 2020.^1^ SARS‐CoV‐2 is an enveloped, single‐stranded RNA virus that emerged at the end of 2019,^2^ after which it spread rapidly.^3^, ^4^ As of April 2021, there have been over 150 million confirmed cases reported to WHO.^5^

The pandemic has resulted in the irreparable loss of millions of lives and an increased burden on health systems. Moreover, it has negatively impacted the economy, education, society, and other sectors. A series of adjustments were formulated rapidly to prevent virus spread, including using masks, improvement of hygiene, and physical distancing measures.^6^ Furthermore, staying at home, changes in transport, and travel patterns have impacted the environment, social interaction, and routine medical practice.

The wide gamut in clinical features,^7^, ^8^ uncertainty in optimal management, and prognosis have posed a significant challenge to the health care community. Meanwhile, health care professionals (HCPs) have focused their efforts on fighting the virus and on exhaustive research, with remarkable advances such as developing vaccinations in a relatively short period. Previous studies have described risk factors for severe SARS‐CoV‐2 infection,^9^ mainly identifying male gender, elderly age, and comorbidities such as hypertension, diabetes, obesity, and others.^10^, ^11^, ^12^

Allergic diseases are highly prevalent and result in significant morbidity and financial burden. Thus, optimizing the care of allergic patients during the pandemic is of uttermost importance. Accordingly, guidelines for allergic conditions were adapted, and a series of international expert panel/consensus documents had developed.^13^, ^14^, ^15^, ^16^ The European Academy of Allergy and Clinical Immunology (EAACI) has promptly released quality information and elaborated position papers and clinical recommendations for allergic disease care.^17^, ^18^, ^19^, ^20^, ^21^, ^22^, ^23^, ^24^

Implementing remote care was one of the most relevant recommendations proposed by international societies^14^ ^,^ ^20^ ^,^ ^25^ to assess disease control, promote patients' compliance, oversee self‐administration of biologics, and provide patient education.^26^ However, these remote tools are not exempt from limitations since there may be regional disparities in implementation and access. Furthermore, its effectiveness in severe conditions is limited.

As a general rule, medical consensus and guidelines are developed based on quality evidence, but given the nature of the pandemic, issuing recommendations supported by prior evidence was not feasible.

Considering the urgent need to assess the impact of the COVID‐19 pandemic on the care of allergic patients, EAACI developed an international survey to generate real‐life experience data. Focusing on practical considerations, the aim was to provide information that could give rise to future management, and recommendations, even out of the pandemic.

## 2 METHODS

The EAACI Immunotherapy Interest group members, alongside other professionals with valuable expertise on allergic patients', developed a 42‐question survey on practical considerations during COVID‐19. The questionnaire was available through an online platform (SurveyMonkey®) to reach out to HCPs providing care to allergic patients worldwide. The survey was disseminated through social media, medical societies, emailed to EAACI members, and made available from February 9 to March 31, 2021, recording responses directly to a strictly anonymized database. Questions were grouped into six domains: (I) General information (Q1–Q6), (II) Allergy practice during COVID‐19 pandemic (Q7–Q10, Q13, and Q26–28), (III) SARS‐CoV‐2 Screening Methods (Q11–Q12, Q24), (IV) Allergy practical considerations and general management during COVID‐19 pandemic related to atopic diseases, treatments, and measures (Q14–Q23, Q25, and Q29–Q36), (V) Planned care of allergic patients after pandemic (Q37), (VI) Allergic patients' management, and COVID‐19 vaccination (Q38–Q41). Question 42, corresponded to additional comments.

## 3 RESULTS

### 3.1 Domain I—“General information (Q1–Q6)”

The survey was answered by 618 HCPs. The majority were allergists (35.4%)/pediatric allergists (27.8%). General information and distribution of respondents' by specialty and place of work are presented in Table S1. Most respondents were from Italy (13.9%), Spain (13.3%), Germany (8.7%), Turkey (5.8%), and Portugal (5.7%), and the rest from 70 countries all over the world (less than 4% each). Europe was the region with the highest number of participants (83%) (Table S2).

### 3.2 Domain II—Allergy practice during COVID‐19 pandemic (Q7–Q10, Q13, Q26–Q28)

**FIGURE 1:**
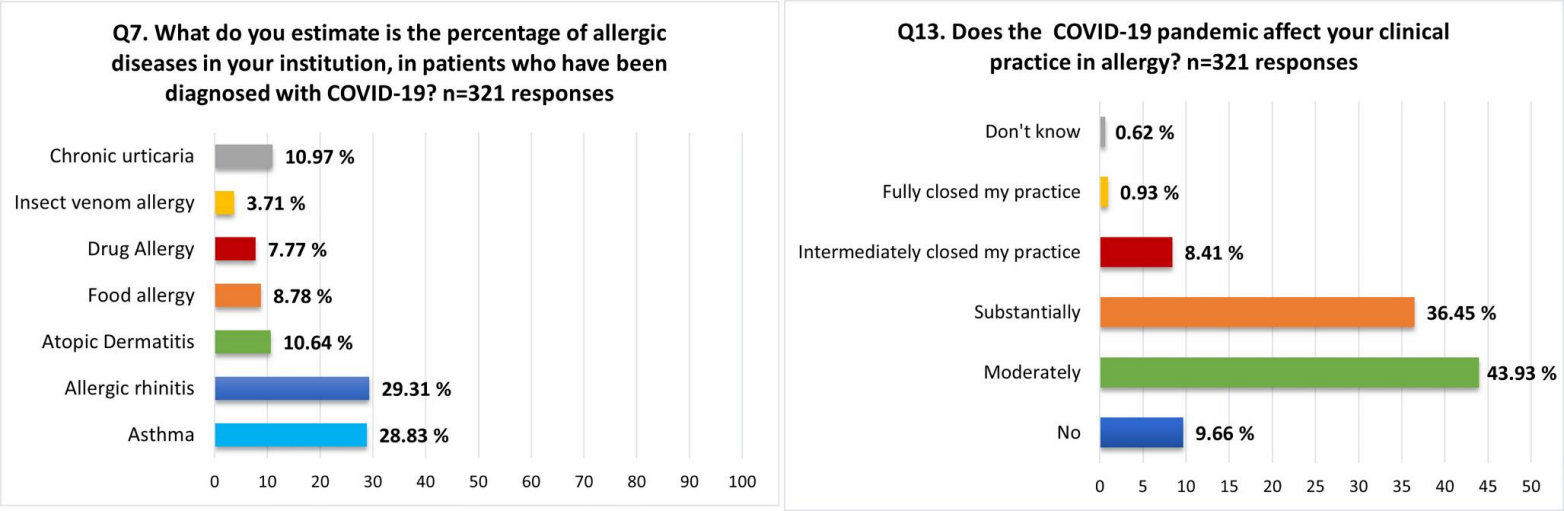
Domain II. Allergy practice during COVID‐19 pandemic. Percentages of responses to Q7 and Q13

The most commonly reported conditions in allergic patients diagnosed with COVID‐19 were asthma and allergic rhinitis (Figure 1, Q7). About 80.4% of HCPs indicated being affected moderately or substantially in their allergy practice during the pandemic, while only a minority (0.9%) needed to completely suspend it (Figure 1, Q13).

Additionally, 27 professionals intermediately closed their practice; 14/27 (51.8%) worked in national health care locations; 9/27 (33.3%) in private practice, and 4/27 (14.8%) in both settings. However, there is no information on whether the practice interruption occurred exclusively due to legislation.

A reduction in different scales of face‐to‐face visits was reported by 93.1%, while 5.6% outlined not having to reduce in‐person care (Figure S1, Q8). The magnitude of the decrease in face‐to‐face care because of patients' personal or doctors'/hospital decisions is shown in Figure S1, Q9.

In‐person visits were maintained by almost 60% of HCPs for patients with severe uncontrolled asthma and almost half for anaphylaxis (47.7%) and less frequently in other conditions. Remarkably, 29.6% of physicians kept performing all visits in person (Figure S1, Q10).

Concerning diagnostic challenge tests, 37.2% continued to carry out all types of challenge tests, whereas 22.9% completely stopped their performance. Nearly 39% suspended only respiratory challenges (nasal, bronchial, and chambers); 5.6% stopped only food challenges, and 3% exclusively interrupted drug challenges (Figure S1, Q26).

About 65% of participants utilized EAACI resources to inform their patients during the pandemic, while 23.9% used published guidelines coming from other organizations (Q27).

HCPs reported their patients' perceptions regarding protective measures during the pandemic, such as using masks. About 57.1% highlighted that its use reduces pollen exposure, thus preventing symptoms. On the contrary, 35.5% indicated improper air exchange, therefore, increasing their symptoms. Additionally, 36.5% reported that their patients' allergic symptoms had been mistakenly interpreted as a viral infection. Finally, 50.5% pointed out a significant patients' fear of infection resulting in fewer visits to allergy facilities (Q28).

### 3.3 Domain III—SARS‐CoV‐2 screening methods (Q11–Q12, Q24)

Most HCPs performed a triage questionnaire prior to face‐to‐face visits, 19.0% required a negative SARS‐CoV‐2 test exclusively for patients who needed spirometry and in procedures with aerosolization high risk (Table 1). About the latter, spirometry, exercise tests, and using peak flow meter devices were considered high‐risk procedures by 92.0%, 67.6%, and 66.0% of the surveyed physicians, respectively (Q24). Finally, 6.8% responded having as a mandatory requirement for in‐person care a negative SARS‐CoV‐2 test. SARS‐CoV‐2 testing time before face‐to‐face visits is displayed in Table 1.

**TABLE 1:** SARS‐CoV‐2 screening methods (Q11–Q12, Q24)

	*n* = 321 responses	%
Q11. Did/Do you perform (or ask your patients) to bring a negative SARS‐CoV‐2 test for patients requiring face‐to‐face visits before consultation?		
Yes, to all	22	6.85
No, only a pre‐visit triage questionnaire was/is performed	238	74.15
Yes, but only in patients who needed spirometry or other high‐risk procedures (spread by aerosolization)	61	19.00

	*n* = 116 responses	%
Q12. If the answer was “yes,” how much time before the visit was the SARS‐CoV‐2 testing performed?		
24 h	29	25.00
48 h	43	37.07
72 h	30	25.86
>72 h	14	12.07

	*n* = 300 responses	%
Q24. In your opinion, which of the following allergy practices increase the risk of COVID‐19 infection spread by aerosolization? You may choose more than one option below		
Spirometry test	276	92.00
Peak flow meter device	198	66.00
Exercise test	203	67.67
Food or drug challenge tests	23	7.67
Skin prick tests	12	4.00
Oral immunotherapy	9	3.00
Allergen immunotherapy for inhalant allergy	7	2.33
Insect venom immunotherapy	4	1.33
Don’t know	11	3.67

Abbreviations: COVID‐19, Coronavirus disease 2019; SARS‐CoV‐2, severe acute respiratory syndrome coronavirus 2.

### 3.4 Domain IV—Allergy practical considerations and general management during COVID‐19 pandemic as related to atopic diseases, treatments, and measures (Q14–Q23, Q25, Q29–Q36)

#### 3.4.1 Asthma (Q14, Q16, Q17, Q19)

Most of the HCPs (81.4%) indicated maintaining an unaltered inhaled corticosteroids (ICS) prescription, while 11.9% increased it. In addition, 2.5% reported a reduction in these prescriptions (Table 2).

**TABLE 2:** Allergy practical considerations and general management during COVID‐19 pandemic in asthmatics patients (Q14, Q16, Q17, Q19)

	*n* = 242 responses	%
Q14. Have you changed your prescriptions of inhaled corticosteroids in asthma patients?		
Yes, I increased	29	11.98
Reduced	6	2.48
I did not change	197	81.40
I do not know	10	4.13

	*n* = 309 responses	%
Q16. If there is a suspicion of COVID‐19 infection, do you continue inhaled steroids at the same dose in asthma patients?		
Yes, I continue inhaled steroids at the same dose	227	73.46
I decrease dose to two third	1	0.32
I decrease dose to half	1	0.32
I stop inhaled steroids	5	1.62
I prefer to use a combination of inhaled steroids with a long‐acting beta‐adrenoreceptor agonist	48	15.54
I do not know	27	8.74

	*n* = 309 responses	%
Q17. If there is a suspicion of COVID‐19 infection, do you continue oral steroids at the same dose in asthma patients?		
Yes, I continue oral steroids at the same dose	173	55.99
I decrease the dose to two third	9	2.91
I decrease the dose to half	22	7.12
I stop oral steroids	25	8.09
I do not know	80	25.89

	*n* = 309 responses	%
Q19. How has the pandemic changed your practice to start biologicals for the treatment of asthma?		
It did not change my practice	150	48.54
Not started new treatment but continued the previously started ones	74	23.95
Stopped the previously started ones	3	0.97
Not applicable/not using biologicals in practice	82	26.54

Abbreviation: COVID‐19, Coronavirus disease 2019.

Regarding, biological treatment initiation, almost half (48.5%) of HCPs kept prescription patterns unaltered. However, about one quarter (23.9%) suspended the start of new treatments, keeping those that were started before the pandemic. Of note, 26.5% outlined not using biologicals in practice, whereas three physicians indicated an interruption of previous treatments (Table 2).

In asthmatic patients with suspected COVID‐19 infection, three out of four physicians maintained ICS at the same dose; 15.5% preferred the prescription of ICS combined with a long‐acting‐beta‐adrenergic agonist. Only a minority (1.6%) discontinued ICS therapy. Regarding oral corticosteroids (OCS) in patients with suspected COVID‐19 infection, 55.9% continued the dose unchanged; 10.0% reduced the OCS dose, while 8.1% discontinued its administration (Table 2).

#### 3.4.2 Allergic rhinitis (Q15, Q18)

The majority (89.9%) of practitioners did not modify intranasal corticosteroids (INCS) prescription behavior, whereas an increase was stated by 5.8%. In patients with allergic rhinitis and suspicion of COVID‐19 infection, five out of six respondents maintained INCS dose unchanged and near 8% interrupted treatment (Table 3).

**TABLE 3:** Allergy practical considerations and general management during COVID‐19 pandemic in allergic rhinitis patients (Q15, Q18)

	*n* = 309 responses	%
Q15. Have you changed your prescriptions of intranasal corticosteroids in patients with allergic rhinitis?		
Yes, I increased the prescriptions	18	5.83
Yes, I reduced the prescriptions	5	1.62
I did not change my prescription patterns	278	89.97
I do not know	8	2.59

	*n* = 309 responses	%
Q18. If there is a suspicion of COVID‐19 infection, do you continue intranasal steroids at the same dose in rhinitis patients?		
Yes, I continued same dose	258	83.50
I decrease the dose to two third	1	0.32
I decrease the dose to half	4	1.29
I stop intranasal steroids	23	7.44
I do not know	23	7.44

Abbreviation: COVID‐19, Coronavirus disease 2019.

##### Allergen immunotherapy for respiratory allergies (Q20, Q25)

Around half (48.9%) continued aeroallergen immunotherapy prescriptions without change, while a reduction in diverse degrees was indicated by 35.6%. Interestingly, 4.2% discontinued prescriptions, and 4.2% changed the route of administration from subcutaneous to sublingual (Table S3).

##### Venom immunotherapy for insect venom allergy (Q21, Q25)

About 60.2% did not change prescription behavior. A reduction in different scales was stated by 9.1%. Of note, 3.9% of HCPs were not prescribing venom immunotherapy (VIT) and 26.9% did not specify otherwise. The question did not discern between treatment initiation and maintenance (Table S3).

##### Allergen immunotherapy for food allergies (Q22, Q23)

Regarding oral immunotherapy (OIT) treatment modifications during the pandemic, 26.9% maintained initiation, 14.9% postponed treatment initiation, and 5.5% entirely suspended it. Regarding the build‐up phase, 20.7% continued performing dose increases, 14.2% conducted increases at longer intervals, and 4.9% suspended them. A high number of HCPs indicated not performing OIT at their practice (Table S3).

#### 3.4.3 Telemedicine (Q29–Q36)

**FIGURE 2:**
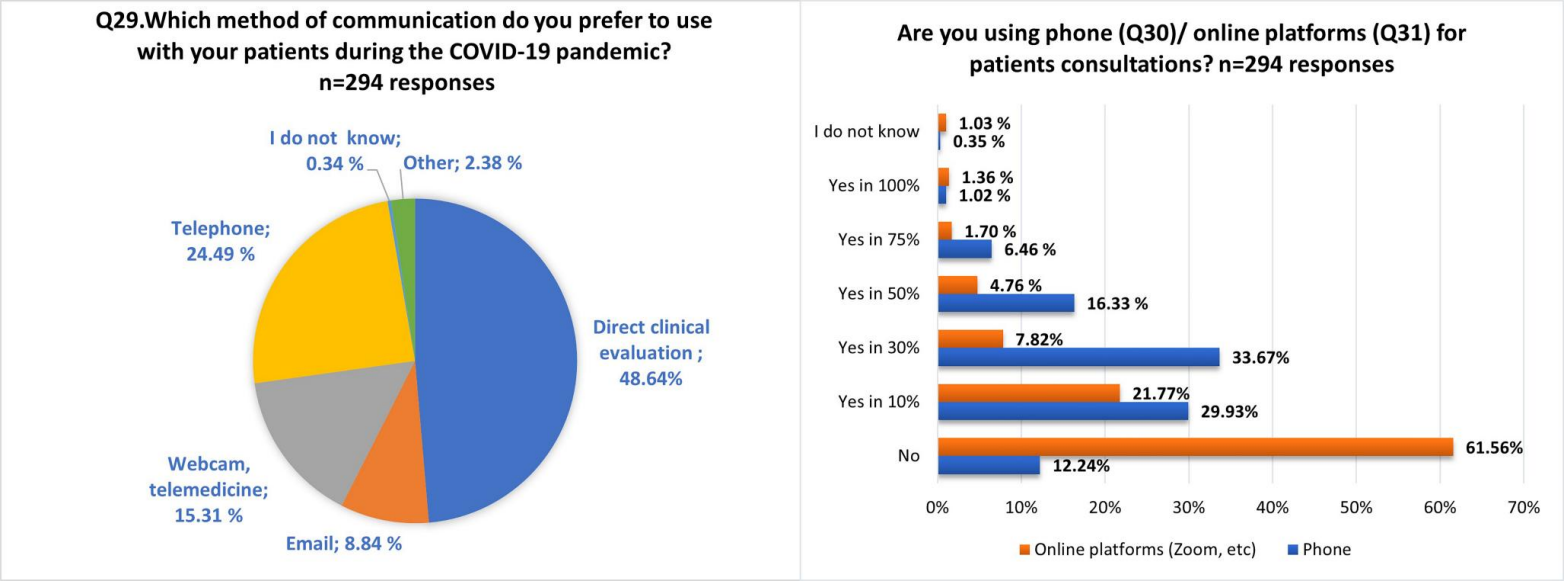
Domain IV, Telemedicine (Q29–Q31)

HCPs more frequently preferred direct clinical evaluation at the hospital/clinic as communication method (48.6%) (Figure 2, Q29). Frequencies of phone and online platforms used for patients' consultations are presented in Figure 2, Q30–Q31.

Interestingly, 42.2% had used telemedicine before the pandemic. In those who had previously used it, 61.1% augmented diversely the number of teleconsultations, whereas 24% did not make modifications in this regard. Concerning participants who had not used telemedicine prior to the COVID‐19 pandemic, 30.5% did not implement new teleconsultation methodologies, while 63.1% increased in a portion of their visits (Table S4).

Half of the respondents pointed out that telemedicine tools had limited efficacy not applicable to all diagnostic scenarios, and the same rate highlighted usefulness in a portion of patients, although consider them not helpful for proper management of severe conditions. Solely, about 9% were satisfied since it allowed them to provide patient care from diagnosis to therapy prescription (Figure S2, Q35).

Data concerning the age range of patients satisfied with telemedicine care are displayed in Table S4. In general, patients under 50 years appeared to be more satisfied.

### 3.5 Domain V—Planned care of allergic patients after pandemic (Q37)

Focusing on future care once the pandemic is over; 55.8% plan to wear protective masks; 40.8% will increase telemedicine use; 33.7% will require maintaining social distancing during visits and use of face masks. Remarkably, 24.1% will carry out their practice in the same manner as before the pandemic.

### 3.6 Domain VI—Allergic patients' management and COVID‐19 vaccination (Q38–Q41)

**FIGURE 3:**
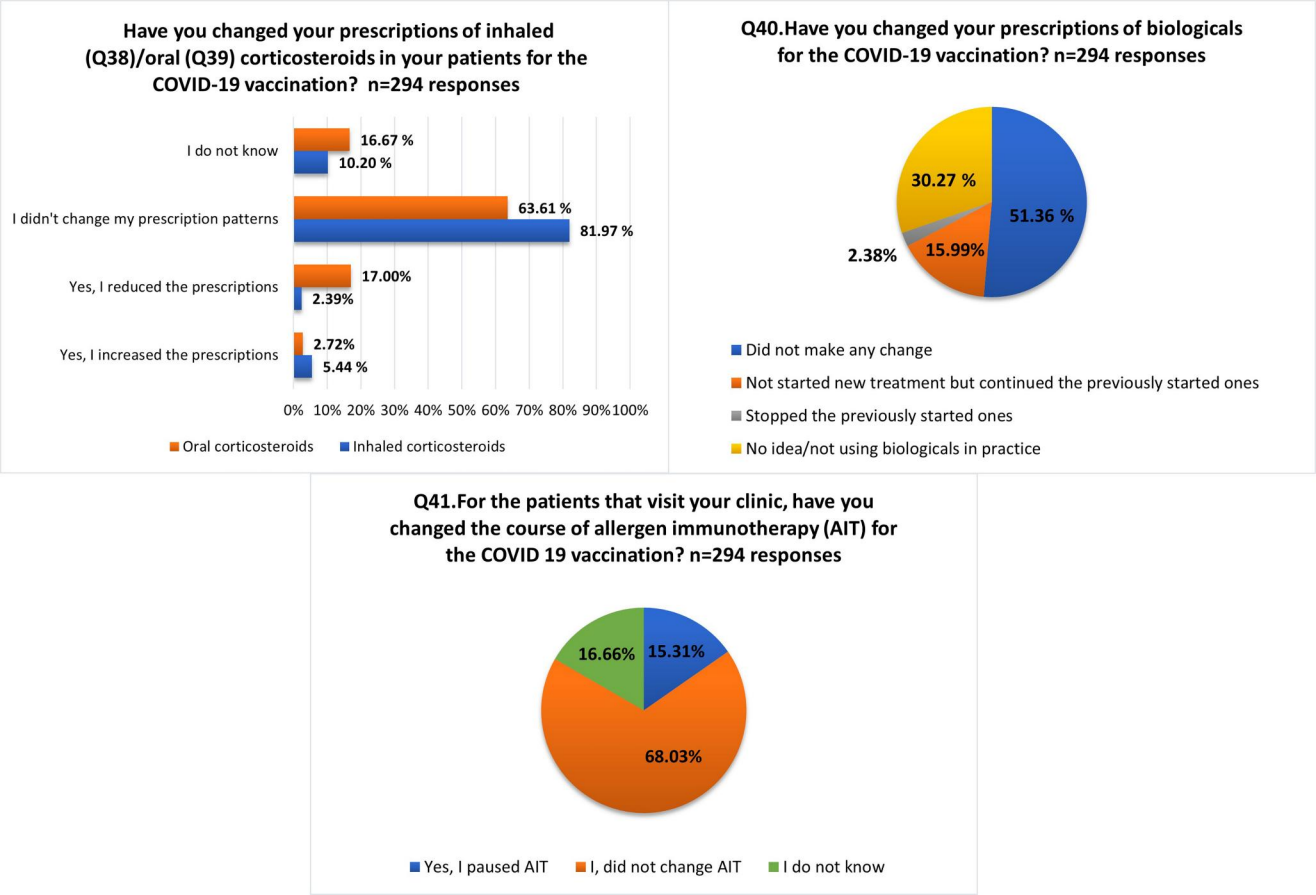
Domain VI: Allergic patients' management and COVID‐19 vaccination (Q38–Q41)

Regarding patients' treatment modifications for the COVID‐19 vaccination, 81.9% did not change ICS prescriptions and 5.4% increased them. Concerning OCS, 63.6% kept the same treatment patterns and 17.0% reduced their prescriptions. 51.4% remained without biological treatment modifications for COVID‐19 vaccination, and 16% did not start new treatments but kept previously started ones. In relation to allergen immunotherapy (AIT), 68.0% made no change in the course of AIT, and 15.3% paused its administration (Figure 3).

## 4 DISCUSSION

A few months after the pandemic declaration, a series of recommendations were issued by leading international scientific societies in the allergy field to provide guidance.^13^ ^,^ ^14^ ^,^ ^16^ ^,^ ^20^ ^,^ ^24^ However, given the COVID‐19 pandemic, such recommendations were formulated rapidly, and as a result of consensus. The pandemic imposed a challenge on HCPs in different medical fields, causing a re‐shaping of the doctor‐patient interaction. The latter was demonstrated by the fact that four out of five HCPs experienced significant impact in their allergy practice. Prioritizing face‐to‐face care for patients with significant in‐person care needs and favoring the use of remote tools in situations that could be managed effectively was recommended by international guidance.^20^ Findings in the present survey (domain II) reflect that almost all practitioners experienced a reduction in face‐to‐face visits.

In‐person care for the management of patients with uncontrolled/severe asthma,^20^ VIT when there is a history of a systemic reaction, food allergy in the context of significant/critical nutritional needs (e.g., infants), anaphylaxis, urgent drug allergy, and severe atopic dermatitis was suggested.^14^ ^,^ ^20^ In line with these international recommendations, clinicians prioritized face‐to‐face visits under such circumstances. Of note, almost a third kept performing all types of visits in person (domain II), comparable to findings from a real‐life international allergy survey.^27^ In a British multicenter study, new urgent consultations were accepted by most allergy services (90%), while a significant reduction in face‐to‐face care was noted. Urgent drug allergy, venom, and food anaphylaxis were prioritized for an in‐person evaluation.^28^

Similarly, in an allergists survey in Turkey, face‐to‐face visits were conducted mainly in patients with anaphylaxis, venom allergy, hereditary angioedema, and drug hypersensitivity.^29^ An Italian pediatric real‐life experience, reported following the regular schedule of face‐to‐face care only patients in critical need for the first evaluation ‬of severe allergic reactions, uncontrolled respiratory allergies, and the ones receiving VIT and biologic treatments.^30^ In a recent North‐American experience, during the first 3 weeks of COVID‐19 restrictions, all food and drug challenges were canceled as well as half of the scheduled visits, while the remaining appointments were more commonly conducted by telephone. Only 2% of face‐to‐face consultations were kept.^31^ It is important to remark that diverse findings may reflect practice disparities influenced by regional differences in imposed restrictions and the constantly changing dynamics of the pandemic over time.^27^

The present pandemic has also impacted diagnostic practice since about one fourth reported a complete suspension of challenge tests while more than one‐third of HCPs continued their performance (domain II). These findings highlight possible delays in the diagnosis and management of some allergic patients. Likewise, a proportion of patients, especially infants, may have experienced a delay in introducing foods to their diet, potentially resulting in a nutritional impact and quality of life impairment. This fact was also noted in a recent pediatric multicenter survey reporting the inability to reintroduce foods as a consequence of not undertaking food challenges.^28^

Domain III investigated SARS‐CoV‐2 screening methods. Respiratory diagnostic challenges, spirometry, and other airway procedures result in aerosol production. Given its inherent spreading risk, a suspension of procedures has been issued. Nonetheless, it is advised to prioritize their performance on a case‐by‐case basis.^20^ Consistently, nine out of 10 HCPs considered spirometry as a high infectious risk procedure, while exercise tests and using peak flow meter devices were outlined as high‐risk but to a lesser extent. However, although these methods were considered high risk, only 19% reported as a prerequisite a negative SARS‐CoV‐2 test to carry them out. Regarding screening practices before face consultations, nearly three‐quarters of HCPs reported conducting just a triage questionnaire (domain III). Similarly, in an allergy practice study, screening for COVID‐19 involved mainly a questionnaire‐based approach, with only 3.2% obtaining SARS‐CoV‐2 tests.^28^

Although some have reported asthma as a relatively frequent comorbidity among patients with COVID‐19,^32^ to date, asthma has not been robustly considered a significant risk factor for developing severe COVID‐19,^33^ increased risk of hospitalization^34^ and mortality.^35^ Furthermore, data from a multinational cohort revealed an improvement in control and outlined that asthmatic children were not disproportionately affected by SARS‐CoV‐2 infection. Such findings were also consistent with the results of an online survey.^36^, ^37^ Published data have not found allergic diseases to be a risk factor for the development nor the severity of SARS‐CoV‐2 infection.^8^ ^,^ ^38^, ^39^

Domain IV in our survey aimed to investigate practical management of atopic diseases, treatment, and measures taken during the ongoing pandemic. In allergic airway diseases, adequate control is paramount to reduce exacerbations. Thus, early recommendations advised not to step down controller medications unless there was a direct benefit on an individualized basis.^14^, ^20^ In keeping with this, most of the HCPs maintained asthmatic patients' prescriptions of ICS unaltered. Focusing on asthmatic patients with suspected COVID‐19 infection, a similar number maintained ICS at the same dose. Concerning OCS in asthmatic patients with suspected COVID‐19 infection, more than half continued the prescribed dose unchanged. There has been a concern about corticosteroid effects on the outcomes of COVID‐19 infection, owing to conflicting evidence suggesting altered immune responses and delay in virus clearance.^40^ GINA 2021 latest report, advised on asthma management during the COVID‐19 pandemic, to maintain controller medications “particularly inhaled corticosteroid‐containing medications and oral corticosteroids if prescribed.”^41^

As regards to biologics, international statements issued to continue their administration in non‐infected patients during the pandemic. Moreover, it was suggested, changing to home administration, after an exhaustive assessment and follow‐up.^14^, ^17^ For the treatment of asthma, almost half of respondents' kept the prescription of biologics initiations in the same manner as before the pandemic. An interruption of biologicals could lead to a worsening of the underlying disease.^20^ Current evidence is lacking suggesting an impaired immune response to COVID‐19 in patients with asthma treated with biologics targeting type 2 inflammation.^17^ Moreover, some preliminary reports suggest that continuing biologics appear to be safe yet withholding treatment if active COVID‐19 infection occurs. Asthmatic viral‐induced exacerbations in patients undertaking biological treatment appear to be less severe and to occur less commonly.^42^, ^43^

Concerning the care of patients with allergic rhinitis, almost all HCPs did not change INCS prescription patterns, and a similar attitude was performed if there was a suspicion of COVID‐19 infection in line with an ARIA‐EAACI statement.^21^

AIT is one of the most important treatments for Immunoglobulin E (IgE)‐mediated allergies as it is the only disease‐modifying therapy.^19^, ^44^ An early statement advised not to initiate AIT during the pandemic for patients with allergic rhinitis unless there is an “unavoidable exposure that has resulted in anaphylaxis or asthma‐related hospitalization.”^14^ Moreover, the continuation of both subcutaneous and sublingual immunotherapy was suggested in non‐infected patients.^19^ Our findings revealed that just a few HCPs completely stopped prescribing AIT for respiratory allergies (4%) during the pandemic and a minority changed the route of administration. About one‐third reduced AIT prescription. However, this query did not allow discrimination between treatment initiation and maintenance. Furthermore, interruption of AIT was discouraged, especially in potentially life‐threatening allergies, such as venom allergy,^19^ and in our survey, more than half did not make any VIT prescription changes. In a recent EAACI survey, almost 60% of respondents indicated not initiating AIT for respiratory allergies, while 16% switched the route from SCIT to SLIT. Moreover, VIT initiation was postponed by 40% of surveyed HCPs.^45^ Other reports in the UK, Portugal, Germany, Austria, and Switzerland have described a significant reduction in VIT initiation during the COVID‐19 pandemic.^28^ ^,^ ^46^, ^47^

Early recommendations advised postponing OIT treatment initiation and up‐dosing until normal practice restoration.^14^, ^16^ Our findings outline that oral immunotherapy (OIT) treatment initiation and dose escalation were continued in some cases. One quarter continued initiating treatment and one‐fifth continued up‐dosing. However, a high number of participants indicated not performing OIT before the pandemic.

Telehealth was encouraged considering its potential to provide remote care while assisting in physical distancing. Allergy and immunology clinicians have needed to adopt telemedicine expeditiously.^26^ ^,^ ^30^ In a systematic review and meta‐analysis before the pandemic, combined‐telemedicine was outlined as an effective intervention for assessing and improving asthma control and patients' quality of life.^48^ Also, it has been a valuable tool for providing asthma education.^49^ High patient satisfaction with telemedicine encounters in allergy/immunology practice has been reported during the COVID‐19 pandemic^50^ and in previous reports.^51^, ^52^

Although there seems to be an apparent increase in telemedicine use worldwide since the onset of the COVID‐19 pandemic, almost half of the practitioners selected direct clinical evaluation at the hospital/clinic as the preferred communication method. Half of the respondents pointed out that telemedicine tools did not apply to all diagnostic scenarios and the same rate noted that these tools worked for some of the patients but were not helpful for patients with severe conditions. Only a minority of HCPs were satisfied with telemedicine tools.

Regarding controller treatment modifications for the COVID‐19 vaccination, the majority did not make any change in the ICS prescription. Also, most clinicians did not make any changes in biological agent prescriptions for planned COVID‐19 vaccination (domain VI). Concerning this matter, a recent consensus report of the German learned societies recommended the treatment and continuation of biologics (allergies and type‐2 inflammation indication) during current COVID‐19 vaccinations, but also emphasized a timely interval between COVID‐19 vaccination and the application of the biologicals.^53^ Indeed, immunosuppressive or immunomodulatory therapies, including biologics, are not contraindicated for COVID‐19 vaccination.^53^

## 5 CONCLUSIONS

The COVID‐19 pandemic has presented an unexpected number of challenges and paradigms to HCPs worldwide. The present EAACI Task Force project pursued to assess the impact of the COVID‐9 pandemic on routine clinical care of allergic patients focusing on practical considerations. The survey provides real‐life results on the consequences experienced in allergy practice. Results indicate a marked in‐person care reduction and a significant suspension in diagnostic challenges, which may affect the care of allergic patients. In keeping with international recommendations, most HCPs prioritized face‐to‐face care only for patients with severe conditions. Furthermore, most respondents maintained unaltered controller treatments for both asthma and allergic rhinitis. Regarding AIT, again, most professionals kept ongoing treatments unchanged. New prescriptions of biologic agents were reduced. Taking into account the challenges faced during the COVID‐19 pandemic, remote care may have resulted in a valuable tool in delivering allergy services during these cumbersome times. The present results could potentially give rise to future recommendations for professionals taking care of allergic patients.

## CONFLICT OF INTEREST

The authors declare that they have no competing interests.

## Supporting information

Supporting Information S1

Supporting Information S2
