# Supplementary material for: Allergic patients during the COVID‐19 pandemic—Clinical practical considerations: An European Academy of Allergy and Clinical Immunology survey
Source: Clin Transl Allergy. 2022 Jan 17;12(1):e12097. doi: 10.1002/clt2.12097 (PMC8762981; doi:10.1002/clt2.12097)
Supplement: Supplementary file 1 — Supporting Information S1 [file CLT2-12-e12097-s002.docx]

Figure S1. Domain II. Allergy practice during COVID-19 pandemic (Q8-10, Q26)

Figure S2. Domain IV, Telemedicine (Q35)
