# Supplementary material for: Allergic patients during the COVID‐19 pandemic—Clinical practical considerations: An European Academy of Allergy and Clinical Immunology survey
Source: Clin Transl Allergy. 2022 Jan 17;12(1):e12097. doi: 10.1002/clt2.12097 (PMC8762981; doi:10.1002/clt2.12097)
Supplement: Supplementary file 2 — Supporting Information S2 [file CLT2-12-e12097-s001.docx]

|  | *n=618 responses* | % |
| --- | --- | --- |
| Q1. Age | | |
| *< 30 years* | 50 | 8.10 |
| *31-45 years* | 250 | 40.45 |
| *45-60 years* | 223 | 36.08 |
| *>60 years* | 95 | 15.37 |
| Q2. Gender | | |
| *Female* | 390 | 63.10 |
| *Male* | 228 | 36.90 |
| Q4. Specialty | | |
| *Allergy* | 219 | 35.44 |
| *Pediatric Allergy* | 172 | 27.83 |
| *Pediatrician* | 54 | 8.74 |
| *ENT* | 46 | 7.45 |
| *Pulmonology* | 33 | 5.34 |
| *Dermatology* | 30 | 4.85 |
| *Immunology/Clinical Immunology* | 24 | 3.88 |
| *Allied Health Professional* | 6 | 0.97 |
| *General Practitioner* | 4 | 0.65 |
| *Others* | 30 | 4.85 |
| Q5. Years of professional experience | | |
| *<1 year* | 15 | 2.43 |
| *1-5 years* | 100 | 16.18 |
| *5-10 years* | 112 | 18.12 |
| *10-20 years* | 140 | 22.65 |
| *>20 years* | 251 | 40.62 |
| Q6. Place of work | | |
| *Private practice* | 167 | 27.02 |
| *National health service, primary care* | 29 | 4.69 |
| *National health service, secondary/tertiary health care settings* | 360 | 58.25 |
| *Private practice and national health primary care service* | 6 | 0.97 |
| *Private practice and national health secondary/tertiary service* | 46 | 7.44 |
| *Other* | 10 | 1.63 |

Table S1. Domain I, General Information (Q1-2, Q4-6).

Abbreviation: *ENT, Ear Nose Throat.*

Table S2. Domain I, General information (Q3): Distribution by region and countries with the highest number of respondents’.

|  | n*=618* | *%* |
| --- | --- | --- |
| Q3. Country of employment | Respondents’ by region |  |
| *Europe* | *511* | *82.69* |
| *America* | *59* | *9.55* |
| *South East Asia* | *14* | *2.26* |
| *Eastern Mediterranean* | *11* | *1.78* |
| *Western Pacific* | *11* | *1.78* |
| *Africa* | *3* | *0.48* |
| *Unspecified* | *9* | *1.46* |
| Countries with the highest number of respondents’ |  |  |
| *Italy* | *86* | *13.91* |
| *Spain* | *82* | *13.27* |
| *Germany* | *54* | *8.74* |
| *Turkey* | *36* | *5.82* |
| *Portugal* | *35* | *5.66* |

|  | *n=309 responses* | *%* |
| --- | --- | --- |
| Q20. Has the prescription of allergen immunotherapy (AIT) for respiratory allergy changed? | | |
| *No, not at all* | *151* | *48.87* |
| *Yes, I am prescribing 25% less* | *52* | *16.83* |
| *Yes, I am prescribing 50% less* | *35* | *11.33* |
| *Yes, I am prescribing 75% less* | *23* | *7.44* |
| *Yes, I am no more prescribing AIT during the pandemic* | *13* | *4.21* |
| *Yes, I am switching all my SCIT prescriptions into SLIT* | *13* | *4.21* |
| *I do not know* | *22* | *7.12* |
| Q21. For the patients that visit your clinic, has the prescription of insect venom immunotherapy (VIT) changed since the COVID-19 pandemic? | | |
| *No, not at all* | *186* | *60.19* |
| *Yes, I am prescribing 25% less* | *13* | *4.21* |
| *Yes, I am prescribing 50% less* | *9* | *2.91* |
| *Yes, I am prescribing 75% less* | *6* | *1.94* |
| *Yes, I am not prescribing* | *12* | *3.88* |
| *I do not know* | *83* | *26.86* |
| Q22. Has the prescription of OIT initiation for food allergy changed? | | |
| *No, I continued to initiate OIT* | *83* | *26.86* |
| *Yes, I stopped initiating OIT* | *17* | *5.50* |
| *Yes, I postponed the initiation of OIT* | *46* | *14.89* |
| *I do not perform OIT* | *163* | *52.75* |
| Q23. Has the increase phase of (OIT) for food allergy changed? | | |
| *No, I continued to increase OIT doses* | *64* | *20.71* |
| *Yes, I stopped OIT increases* | *15* | *4.85* |
| *Yes, the interval visits at the increase phase are longer* | *44* | *14.24* |
| *I do not perform OIT* | *186* | *60.19* |
| Q25. What type of allergen immunotherapy do your patients receive? | | |
| *Subcutaneous immunotherapy (aeroallergens) (SCIT)* | *214/301* | *71.10* |
| *Subcutaneous immunotherapy to bee/wasp (SCIT)* | *141/301* | *46.84* |
| *Sublingual immunotherapy (SLIT)* | *234/301* | *77.74* |
| *I do not know* | *17/301* | *5.65* |

Table S3. Domain IV, Allergy practical considerations and general management during COVID-19 pandemic: Allergen-specific Immunotherapy (Q20-Q23, Q25).

*Abbreviations: COVID-19, Coronavirus disease 2019; AIT, allergen-specific immunotherapy; OIT, oral immunotherapy; VIT, venom immunotherapy.*

Table S4. Domain IV, Allergy practical considerations and general management during COVID-19 pandemic: Telemedicine (Q32-34, Q36).

|  | *n=294 responses* | *%* |
| --- | --- | --- |
| Q32. Were you using telemedicine before the COVID-19 pandemic? | | |
| *Yes* | *43* | *14.63* |
| *No* | *170* | *57.82* |
| *Occasionally* | *81* | *27.55* |
| *I do not know* | *0* | *0* |
|  | ***n=224 responses*** | ***%*** |
| Q33. If yes, have you raised the number of teleconsultations? | | |
| *No* | *54* | *24.11* |
| *Yes, in 10% of patients* | *37* | *16.52* |
| *Yes, in 30% of patients* | *47* | *20.98* |
| *Yes, in 50% of patients* | *33* | *14.73* |
| *Yes, in 70% of patients* | *17* | *7.59* |
| *Yes, in 100% of patients* | *3* | *1.34* |
| *Yes, but only during lockdown* | *13* | *5.80* |
| *I do not know* | *20* | *8.93* |
|  | ***n=236 responses*** | ***%*** |
| Q34. If no, have you implemented new telemedicine methods in your practice (including video-consultations, phone-calls, etc…), and to how many patients? | | |
| *No* | *72* | *30.51* |
| *Yes in 10% of patients* | *65* | *27.54* |
| *Yes, in 30% of patients* | *50* | *21.19* |
| *Yes, in 50% of patients* | *24* | *10.17* |
| *Yes, in 70% of patients* | *10* | *4.24* |
| *Yes, in 100% of patients* | *3* | *1.27* |
| *I do not know* | *12* | *5.08* |
|  | ***n=294 responses*** | ***%*** |
| *Q36.* If you are only visiting adult patients, what age range of patients feel most satisfied *with telemedicine?* | | |
| *Under 30 years* | *53* | *18.03* |
| *Under 40 years* | *49* | *16.67* |
| *Under 50 years* | *39* | *13.26* |
| *Under 60 years* | *21* | *7.14* |
| *Under 70 years* | *11* | *3.74* |
| *I do not know* | *121* | *41.16* |

Abbreviations; *COVID-19, Coronavirus disease 2019*
